# Editorial: Community series in novel insights into immunotherapy targeting tumor microenvironment in ovarian cancer: volume I

**DOI:** 10.3389/fimmu.2023.1192190

**Published:** 2023-04-11

**Authors:** Xiang-jie Di, Qi Zhao, Hai-tao Wang, Xia-wei Wei, Xiao Liang

**Affiliations:** ^1^ Key Laboratory of Obstetrics & Gynecologic and Pediatric Diseases and Birth Defects of Ministry of Education, West China Second Hospital, Sichuan University, Chengdu, Sichuan, China; ^2^ Laboratory of Aging Research and Cancer Drug Target, State Key Laboratory of Biotherapy and Cancer Center, National Clinical Research Center for Geriatrics, Clinical Trial Center/NMPA Key Laboratory for Clinical Research and Evaluation of Innovative Drug, West China Hospital, Sichuan University, Chengdu, Sichuan, China; ^3^ Ministry of Education (MoE) Frontiers Science Center for Precision Oncology, Cancer Center, Faculty of Health Sciences, University of Macau, Macau, Macau SAR, China; ^4^ Thoracic Surgery Branch, Center for Cancer Research, National Cancer Institute (NCI), Bethesda, MD, United States

**Keywords:** ovarian cancer, tumor immune microenvironment, immunotherapy, exosomes, non-tumor cells targeting

Ovarian cancer (OC) is a lethal gynecologic malignancy with an extremely low 5-year survival rate of approximately 50% (1). Although technological advancements have led to a decline in OC mortality over the past decade, it still remains unsatisfactory (2). In addition to traditional treatments such as surgery, radiotherapy, and chemotherapy, immunotherapy has made an indelible mark in the field of anti-tumor therapy (3), particularly as tumor microenvironment (TME) is proposed as an important factor in the initiation and progression of ovarian cancer. Immunotherapies targeting TME seems to be promising approach for OC treatment (4). In this Research Topic, we gathered 12 articles to providing us in-depth evaluations of immunotherapies targeting the dysfunctional immune cells, malignant stroma cells, tumor-promoting soluble factors, and exosomes in the OC microenvironment.

The rapid development of sequencing technology has provided a new and convenient way for us to investigate the molecular and immune changes in OC. In the first article of this topic, Ren et al. used single-cell sequencing to integrate a comprehensive cellular and immunological analysis using paired ascites, tumor and peripheral blood samples. The authors highlighted the key role of immunosuppressive cells, including MDSCs, γδT cells, along with CD8+ effector T cells, in recurrence and chemoresistance ascites. The study also emphasized the chemotherapy induced clonal expansion change of TCR/BCR in peripheral blood. These findings aid to develop new immune-modulatory strategies for patients with relapses or chemo-resistant OC.

OC is usually associated with local and distant metastases, rendering a systemic disease with functional and compositional changes in immune system. Thus, the review provided by Rajtak et al. stands apart from other researchers, as they integrated both local and systemic immunity in OC immunotherapy. The authors raised several points for our attention, including the lack of a large patient cohort both locally and systemically, and mechanisms of tumor cell circulation will be studied in the future.

Besides immune cells, dysfunctional malignant stroma cells and tumor-promoting soluble factors are important therapeutic targets in TME and have attracted great research and clinical interest (5). Novel cancer immunotherapy insights were mined from big data by Chen et al. They figured out a set of potential immunotherapeutic target genes in OC. Single-cell sequencing data shows that some of these target genes are mainly expressed in cancer-associated fibroblasts (CAFs), which are tightly associated with the immunotherapy response of OC patients. This study provides new insights into OC immunotherapy. However, it is unfortunate that the population in this study was mainly white. We hope that multiracial study could be given more attention in the future. Another important stroma cell in TME, cancer-associated mesothelial cells (CAMs), is discussed by Zheng et al.. They summarized the key roles of CAMs in OC progression, prognosis and targeting therapy. This review helped with our continued understanding of CAMs in OC and the development of new and effective therapeutic regimens. As a soluble regulator factor in TME, interferons (IFNs) can influence most of the cells in TME. Liu et al. reviewed the multiple effects of IFNs in OC therapy. They proposed that IFNs can assist anti-ovarian cancer therapy by directly affecting the function and survival of tumor cells and immune cells. Based on the summary of the literature, the authors still had confidence in the treatment of OC with IFNs as therapeutic.

Exosomes are extracellular vesicles measuring 30–100 nm, secreted by living cells. As one of the messengers between tumor cells and their surroundings, they have received significant attention in anti-tumor therapy (6). In this Research Topic, Gong et al. comprehensively described the immunotherapy related biomarkers on exosomes isolated from various body fluids of OC patients and reviewed the vital roles of exosomes in OC immunotherapy and diagnosis. In addition, Luo et al. revealed the anti-tumor effects of NK cell-derived exosomes (NK-EXOs) in OC, demonstrating that the anti-tumor activity of NK-EXOs is not only through the high-efficient up-taken of ovarian tumor cells, but also through reversing NK cells immunosuppression in TME. As a natural stability, low immunogenicity, and tumor targeting vector, NK-EXOs can efficiently deliver DDP to ovarian tumor cells and show a great prospect in OC targeting therapy. This research began with clinical isolation and characterization of exosomes and then verified their findings with a variety of laboratory techniques, making this research very solid.

Apart from exosomes, nanoparticles as another small carrier have been expected to play significant roles in immunotherapy. The nanoparticles can not only directly induce tumor cell death, which promotes antigen presentation and immune activation, but also be used as excellent drug delivery system (DDS) to help with targeting immunotherapy agent delivery (7). Various formulations of DDS have been designed to realize the controlled and targeted immunotherapy agent delivery in OC. In this topic, Peng et al. discussed the research and clinical way to modulate OC microenvironment with DDS. The authors described strategies to improve the efficacy of immunotherapy in OC with DDS, especially by targeting TME. Xu et al. focused on the different nanomaterials used in OC immunotherapy and the promising advances they induced in TME modulating.

Immune checkpoint blockade (ICB) therapy has been a popular anticancer treatment strategy for decades and made an indelible mark in tumor immunotherapy. However, resistance and low-response to ICBs restrict their application in OC treatment, and immune-related adverse events (irAEs) complicate treatment (8). In this Research Topic, we received several reviews focusing on clinical ICB therapy. Wang et al. focused on how the TME component, especially immune cells, influenced the ICB therapy response in OC. To avoid irAEs caused by ICB therapy, Xu et al. reviewed the most up-to-date information on prognostic and predictive biomarkers for ICB therapy and gave valuable advice for guiding precision immunotherapy. Hu et al. provided an overview of various clinically oriented forms of multi-immunotherapy in relation to OC and explored possible combinations of immunotherapies that may be effective, which is of utmost importance to OC clinical multi-immunotherapy.

In recent years, significant progress in lab and clinical research has been achieved, although immunotherapies in OC are still being tested in clinical trials. With the help of all these authors in this topic, we have gained a deeper and broader understanding of TME targeting immunotherapy in OC.

## Author contributions

Conceptualization and writing, X-JD and XL; review and editing, H-TW and QZ; supervision, XL and X-WW. All authors have read and agreed to the published version of the manuscript.
